# Mutations in Encephalomyocarditis Virus 3A Protein Uncouple the Dependency of Genome Replication on Host Factors Phosphatidylinositol 4-Kinase IIIα and Oxysterol-Binding Protein

**DOI:** 10.1128/mSphere.00068-16

**Published:** 2016-05-11

**Authors:** Cristina M. Dorobantu, Lucian Albulescu, Heyrhyoung Lyoo, Mirjam van Kampen, Raffaele De Francesco, Volker Lohmann, Christian Harak, Hilde M. van der Schaar, Jeroen R. P. M. Strating, Alexander E. Gorbalenya, Frank J. M. van Kuppeveld

**Affiliations:** a Virology Division, Department of Infectious Diseases and Immunology, Faculty of Veterinary Medicine, Utrecht University, Utrecht, The Netherlands; b Istituto Nazionale Genetica Molecolare “Romeo ed Enrica Invernizzi”, Milan, Italy; c Department of Infectious Diseases, Molecular Virology, University of Heidelberg, Heidelberg, Germany; d Department of Medical Microbiology, Leiden University Medical Center, Leiden, The Netherlands; e Faculty of Bioengineering and Bioinformatics, Lomonosov Moscow State University, Moscow, Russia; Boston University School of Medicine

**Keywords:** EMCV, OSBP, PI4KA, PI4P, cholesterol, mutants, picornavirus, replication organelles, viral resistance

## Abstract

Positive-strand RNA [(+)RNA] viruses are true masters of reprogramming host lipid trafficking and synthesis to support virus genome replication. Via their membrane-associated 3A protein, picornaviruses of the genus *Enterovirus* (e.g., poliovirus, coxsackievirus, and rhinovirus) subvert Golgi complex-localized phosphatidylinositol 4-kinase IIIβ (PI4KB) to generate “replication organelles” (ROs) enriched in phosphatidylinositol 4-phosphate (PI4P). PI4P lipids serve to accumulate oxysterol-binding protein (OSBP), which subsequently transfers cholesterol to the ROs in a PI4P-dependent manner. Single-point mutations in 3A render enteroviruses resistant to both PI4KB and OSBP inhibition, indicating coupled dependency on these host factors. Recently, we showed that encephalomyocarditis virus (EMCV), a picornavirus that belongs to the *Cardiovirus* genus, also builds PI4P/cholesterol-enriched ROs. Like the hepatitis C virus (HCV) of the *Flaviviridae* family, it does so by hijacking the endoplasmic reticulum (ER)-localized phosphatidylinositol 4-kinase IIIα (PI4KA). Here we provide genetic evidence for the critical involvement of EMCV protein 3A in this process*.* Using a genetic screening approach, we selected EMCV mutants with single amino acid substitutions in 3A, which rescued RNA virus replication upon small interfering RNA (siRNA) knockdown or pharmacological inhibition of PI4KA. In the presence of PI4KA inhibitors, the mutants no longer induced PI4P, OSBP, or cholesterol accumulation at ROs, which aggregated into large cytoplasmic clusters*.* In contrast to the enterovirus escape mutants, we observed little if any cross-resistance of EMCV mutants to OSBP inhibitors, indicating an uncoupled level of dependency of their RNA replication on PI4KA and OSBP activities. This report may contribute to a better understanding of the roles of PI4KA and OSBP in membrane modifications induced by (+)RNA viruses.

**IMPORTANCE** Positive-strand RNA viruses modulate lipid homeostasis to generate unique, membranous “replication organelles” (ROs) where viral genome replication takes place. Hepatitis C virus, encephalomyocarditis virus (EMCV), and enteroviruses have convergently evolved to hijack host phosphatidylinositol 4-kinases (PI4Ks), which produce PI4P lipids, to recruit oxysterol-binding protein (OSBP), a PI4P-binding protein that shuttles cholesterol to ROs. Consistent with the proposed coupling between PI4K and OSBP, enterovirus mutants resistant to PI4KB inhibitors are also resistant to OSBP inhibitors. Here, we show that EMCV can replicate without accumulating PI4P/cholesterol at ROs, by acquiring point mutations in nonstructural protein 3A. Remarkably, the mutations conferred resistance to PI4K but not OSBP inhibitors, thereby uncoupling the levels of dependency of EMCV RNA replication on PI4K and OSBP. This work may contribute to a deeper understanding of the roles of PI4K/PI4P and OSBP/cholesterol in membrane modifications induced by positive-strand RNA viruses.

## Abstract (precis)

Positive-strand RNA viruses modulate lipid homeostasis to generate unique, membranous “replication organelles” (ROs) where viral genome replication takes place. Hepatitis C virus, encephalomyocarditis virus (EMCV), and enteroviruses have convergently evolved to hijack host phosphatidylinositol 4-kinases (PI4Ks), which produce PI4P lipids, to recruit oxysterol-binding protein (OSBP), a PI4P-binding protein that shuttles cholesterol to ROs. Consistent with the proposed coupling between PI4K and OSBP, enterovirus mutants resistant to PI4KB inhibitors are also resistant to OSBP inhibitors. Here, we show that EMCV can replicate without accumulating PI4P/cholesterol at ROs, by acquiring point mutations in nonstructural protein 3A. Remarkably, the mutations conferred resistance to PI4K but not OSBP inhibitors, thereby uncoupling the levels of dependency of EMCV RNA replication on PI4K and OSBP. This work may contribute to a deeper understanding of the roles of PI4K/PI4P and OSBP/cholesterol in membrane modifications induced by positive-strand RNA viruses.

## INTRODUCTION

Genome replication of positive-strand RNA [(+)RNA] viruses is tightly associated with virus-induced membranous structures that accumulate in the cytoplasm of infected cells. In the case of hepatitis C virus (HCV), a virus of the *Flaviviridae* family, these structures are referred to as “the membranous web” (MW), whereas for viruses belonging to the *Picornaviridae* family, the most commonly used term is “replication organelles” (ROs) (recently reviewed in references 1 and 2). These virus-induced membranes have been suggested to provide a structural platform that facilitates cooperation between components of the viral replication complex and possibly to also provide shelter from host defense systems (3, 4), but their precise function is not yet understood. Viruses build these specialized membranous structures by drastically rewiring essential cellular processes, especially pathways involved in lipid metabolism.

Picornaviruses manage to efficiently manipulate the cellular environment and transform it into a membranous replication factory using only a few viral nonstructural proteins (5). One of the key viral players involved in this process is the small protein 3A. Picornavirus 3A proteins invariably include a hydrophobic domain at the C terminus but otherwise share little sequence similarity with viruses from different genera, likely due to profound divergence (6). The best-studied picornavirus 3A protein is that of enteroviruses (such as poliovirus [PV; *Enterovirus C* species] and coxsackievirus B3 [CVB3; *Enterovirus B*]). The interaction of 3A with a number of essential host factors results in their accumulation at the membranes of ROs to promote viral RNA synthesis (7–12). Often referred to as “recruitment,” the molecular mechanism behind this accumulation remains unknown and may involve both recruitment and/or “retention” of the host factors. Among the factors that are enriched at enterovirus ROs are the guanine exchange factor GBF1 and the Golgi complex-localized lipid kinase PI4KB (phosphatidylinositol 4-kinase type III isoform β). PI4KB is one of the four mammalian kinases that catalyze the phosphorylation of PI (phosphatidylinositol) to PI4P (phosphatidylinositol 4-phosphate) (13–15). The accumulation of PI4KB at ROs via 3A interaction results in a local increase of the PI4P concentration (8). PI4P was suggested to function in enterovirus replication by promoting downstream processes of RO membrane biogenesis involving essential host factors. Indeed, we and others have recently shown that PI4P is important for the accumulation of oxysterol-binding protein (OSBP) at enterovirus ROs (10, 16, 17). In noninfected cells, OSBP plays a key role in membrane homeostasis by mediating the nonvesicular transport of cholesterol in exchange for PI4P between the endoplasmic reticulum (ER) and Golgi complex (18). OSBP bridges ER and *trans*-Golgi complex membranes at membrane contact sites (MCSs) and shuttles cholesterol from the ER into the Golgi complex and PI4P from the Golgi complex back to the ER, where it is hydrolyzed by the PI4P-phosphatase Sac1. PI4P lipids are critical in this process both by providing the energy required for the countertransport of cholesterol and by serving as a membrane anchor for OSBP. In enterovirus-infected cells, OSBP functions at membrane contact sites (MCSs) between the ROs and ER by shuttling PI4P from the ROs to ER membranes and cholesterol from the ER to ROs, thereby enriching the ROs in cholesterol. To date, why enteroviruses promote cholesterol accumulation at their ROs has remained poorly understood. The lipid transfer function of OSBP is critical for enterovirus replication, since OSBP inhibitors such as OSW-1 and itraconazole (ITZ), which impair this lipid exchange, also inhibit virus genome replication (16). We and others have shown that enteroviruses CVB3 and PV can acquire single-point mutations in their 3A proteins that render them resistant to PI4KB inhibitors (19–23). Remarkably, the same mutations also provide cross-resistance to OSBP inhibitors (16, 24, 25), indicating the coupled dependency of enterovirus replication on PI4KB and OSBP.

Recently, we have also addressed the role of membranes in the replication of another picornavirus, namely, the cardiovirus encephalomyocarditis virus (EMCV, *Cardiovirus A* species), which is closely related to the Theiler’s murine encephalomyelitis virus and the human Saffold virus (both *Cardiovirus B* species) (26). Similarly to what was observed during enterovirus infection, PI4P lipids proved essential for the accumulation of OSBP and cholesterol to the ROs of cardioviruses. We discovered that EMCV induces the formation of PI4P-enriched ROs by hijacking the ER-localized PI4KA (phosphatidylinositol 4-kinase type III isoform α) instead of the Golgi complex-associated PI4KB. Similarly to enteroviruses, and despite little sequence conservation, EMCV seems to employ the viral protein 3A in this process, as revealed by coimmunoprecipitation of PI4KA with 3A from cell lysates and by immunofluorescence (IF) studies showing colocalization of 3A with PI4KA. Thus, enteroviruses and cardioviruses, representing two distantly related picornavirus genera, have evolved to employ their sequence-dissimilar 3A proteins to hijack different host kinases that ensure abundant PI4P production at the ROs. Remarkably, the PI4KA-OSBP pathway used by EMCV is also exploited by HCV to develop a cholesterol-enriched MW (27), suggesting a functional convergence of EMCV and HCV. To date, studies of (viable) HCV mutants resistant to PI4KA inhibitors that could bring novel insights into the role of PI4KA and PI4P in virus replication have been lacking.

In this study, by combining the power of traditional forward genetics with modern techniques of selective protein targeting, reverse genetics, and cell visualization, assisted by bioinformatics, we aimed to gain further insight into the molecular mechanism through which picornaviruses remodel host membranes. To this end, we report the first isolation of (+)RNA viruses that exhibit a markedly decreased dependence on the essential host factor PI4KA. In contrast to the enterovirus mutants described previously, the EMCV mutants were only minimally cross-resistant to OSBP inhibition or depletion, suggesting a remarkable uncoupling of the level of virus replication dependency on PI4KA and OSBP activities. In conclusion, we here reveal that the phenotype of PI4KA-resistant cardioviruses both resembles and differs from that of PI4KB-resistant enteroviruses, with respect to usage of critical host factors for RO membrane biogenesis. Our findings indicate prospects of novel insights into the roles of PI4P and cholesterol in (+)RNA virus replication.

## RESULTS

### EMCV acquires single-point mutations in the 3A gene to restore replication in cells with low levels of PI4KA.

**FIG 1:**
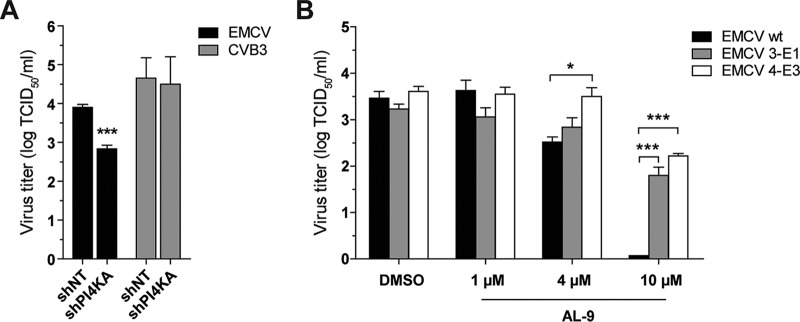
Isolation of EMCV cultures resistant to inhibition of host factor PI4KA. (A) Effects of PI4KA knockdown on EMCV and CVB3 infection. Huh7-Lunet/T7-shNT and Huh7-Lunet/T7-shPI4KA cells were infected with EMCV or CVB3 at MOI 1. After 8 h, cells were freeze-thawed to release intracellular virus particles and the total virus titers were determined by endpoint dilution. (B) Effect of PI4KA inhibitor AL-9 on wt EMCV and EMCV pools 3-E1 and 4-E3. HeLa R19 cells were infected at MOI 1. Total virus titers were determined as described for panel A. Mean values ± standard errors of the means (SEM), following subtraction of the input levels, are shown in both panels. Means were statistically compared using unpaired *t* tests. *, *P* < 0.05; ***, *P* < 0.001.

We set out to isolate mutants of the EMCV species (strain mengovirus) which could replicate in cells with compromised PI4KA activity. To this end, we attempted to propagate wt EMCV in cells in the presence of AL-9, a well-established PI4KA inhibitor (28) that hampers EMCV replication by targeting the enzymatic activity of PI4KA (26). However, all our attempts proved unsuccessful, most likely due to long-term cytotoxic effects of the compound. We then explored another approach that involved propagation of wt EMCV in the stable cell line Huh7-Lunet/T7-shPI4K, in which levels of endogenous PI4KA have been reduced using short hairpin RNA (shRNA)-expressing lentiviral vectors (29). First, we checked whether EMCV replication was impaired in these cells compared to the control cell line Huh7-Lunet/T7-shNT, which expresses a nontargeting shRNA vector. Indeed, the EMCV progeny yield was significantly reduced in the shPI4K cell line compared to the control cell line (Fig. 1A). The observed inhibition was genuine, since the levels of replication of CVB3, which depends on PI4KB rather than PI4KA (8), were similar in the two cell lines (Fig. 1A). Thus, we considered the Huh7-Lunet/T7-shPI4K cell line to be a promising system for the selection of EMCV mutants that are less dependent on PI4KA for replication.

To select EMCV escape mutants, we next infected Huh7-Lunet/T7-shPI4K cells with wt EMCV and harvested individual virus cultures from the wells which developed cytopathic effects (CPE) faster than the others. From these, we selected two virus cultures, named 3E1 and 4E3 after their positions in the plate, which we found to be less sensitive to AL-9 treatment than the wt virus (Fig. 1B). We interpreted this result as indicative of a decreased dependence of the 3E1 and 4E3 mutants on the presence of PI4KA. Genomic analysis of the P2P3 region of these mutants identified two independently acquired single-point mutations, one in each analyzed virus pool: A32V (alanine at position 32 changed to alanine) in 3E1 and A34V (alanine at position 34 changed to valine) in 4E3. No other mutations were found in the sequenced P2P3 region of the two virus pools.

### 3A protein mutations are located in a putative determinant of intermolecular interaction.

**FIG 2:**
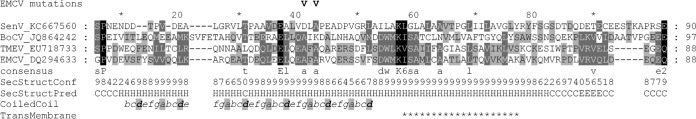
Mutant EMCVs harbor single amino acid substitutions in nonstructural protein 3A. The locations of the acquired mutations in 3A in the context of sequence conservation and putative structural features of this protein are shown. A multiple-sequence alignment (MSA) of 3A of four picornaviruses forming a monophyletic cluster was obtained as specified in Materials and Methods. The name of each MSA entry includes the acronym of the virus name and the GenBank accession number of the respective sequence. SenV, Seneca virus; BoCV, Boone cardiovirus; TMEV, Theiler’s murine encephalomyelitis virus; EMCV, encephalomyocarditis virus. Amino acid conservation is highlighted with shadows using the conserved panel in default mode, which produces a consensus line featuring the invariant residues (uppercase letters) and the most frequent residues (lowercase letters), as well as 6 groups of physicochemically similar residues (digits from 1 to 6). SecStructConf and SecStructPred, confidence and state (C, coil; H, helix; E, strand) of predicted secondary structure of EMCV 3A according to PsiPred analysis of the MSA. CoiledCoil, elements of coiled-coil heptads in EMCV 3A protein as assigned by Paircoil2 with a window length of 21 under the *P* score cutoff of 0.025 (range, 0.0223 to 0.0041). The hydrophobic *a* and *d* positions are contrasted with background shading. Transmembrane residues of EMCV 3A whose data were above the TMHMM2 threshold of 0.4 to form transmembrane helix are indicated with an asterisk (*).

The fact that the two independently acquired substitutions were of the same type, representing changes from Ala to Val, and were accepted at the two positions in close proximity indicated a common mechanism of escape of the mutants. Subsequent bioinformatics analysis of 3A proteins revealed considerable sequence conservation of this protein in viruses of the known three *Cardiovirus* species and one species of the sister *Senecavirus* genus (Fig. 2). According to Psipred v.2.5 results (30), the protein may predominantly adopt the alpha-helix conformation, with the N-terminal part of EMCV (from residue 7 to residue 45) being folded into a left-handed coiled-coil (according to Paircoil2 results [31]) and the C-terminal richly hydrophobic region (from residue 47 to residue 65) forming a transmembrane helix (according to TMHMM2 results [32]). The most conserved amino acid block is located at the junction between the putative coiled-coil and transmembrane structural elements, indicative of considerable constraints and the functional importance of this region. The coiled-coils are alpha-helix oligomers which are distinguished by the presence of several heptad repeats with alternating hydrophilic and hydrophobic residues that are labeled with letters *a* to *g* (33). Residues at the *a* and *d* positions are found at the helix interface, while residues at the other positions are solvent exposed. In line with the canonical coiled-coil organization, hydrophobic and hydrophilic residues dominate at, respectively, the *a* and *d* positions and at the remaining five positions in the aligned 3A proteins (Fig. 2). The A32V and A34V substitutions in the 3A protein of the mutants are located in the *g* and *b* positions of two adjacent heptads, respectively, being partially conserved and among the few exceptionally hydrophobic residues at these positions in all heptads. Accordingly, the mutated residues in 3A may be part of the solvent-exposed coiled-coil surface, which would be compatible with the properties of molecular determinants mediating interaction of 3A with PI4KA directly or indirectly.

### Introduction of the point mutations into the 3A of wt EMCV rescues virus replication from PI4KA inhibition.

**FIG 3:**
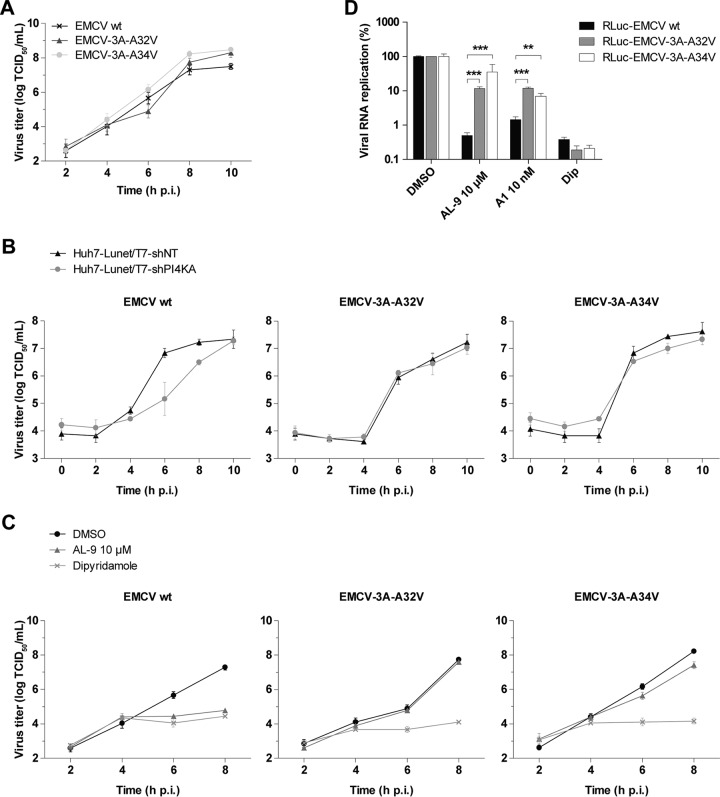
Distinct single-point mutations in 3A render EMCV less dependent on PI4KA. (A to C) Growth curve analysis of EMCV wt and mutant strains. After infection at MOI 1 for 30 min, the inoculum was removed and fresh medium was added to the cells. At the indicated time points, cells were freeze-thawed to determine the total virus titers by endpoint dilution. (A) Growth curves in the absence of compounds in HeLa R19 cells. (B) Growth curves in Huh7-Lunet/T7-shNT and Huh7-Lunet/T7-shPI4KA cells. (C) Growth curves in HeLa R19 cells in the presence of DMSO, 10 µM AL-9, or dipyridamole (Dip). (D) RNA replication of EMCV mutants is resistant to PI4KA inhibitors. HeLa R19 cells were infected at MOI 1 with wt or mutant EMCV reporter viruses carrying the *Renilla* luciferase gene. After 30 min, virus was removed and compound-containing medium was added to the cells. At 8 h p.i., cells were lysed to determine the intracellular luciferase activity. The value for DMSO samples was set at 100%. Mean values ± SEM are shown. Means were statistically compared using unpaired *t* tests. **, *P* < 0.01; ***, *P* < 0.001.

Since we had sequenced only a part of the genome of the EMCV mutants, mutations in other regions might in theory have contributed to the observed phenotype. To exclude this possibility, we introduced the acquired 3A single-point mutations into the full-length infectious clone of wt EMCV, generating the recombinant viruses carrying the individual substitutions: EMCV-3A-A32V and EMCV-3A-A34V. We have also generated the respective luciferase reporter viruses RLuc-EMCV-3A-A32V and RLuc-EMCV-A34V, which contain additionally the gene encoding *Renilla* luciferase upstream of the capsid-coding region. To gain insight into the replication kinetics of the mutant viruses compared to that of the wt virus, single-cycle replication assays were performed. Both mutant viruses replicated similarly to the wt (Fig. 3A), demonstrating that the point mutations in 3A did not affect the kinetics of virus replication under standard conditions. Subsequently, we investigated the replication kinetics of the mutants relative to that of wt virus under conditions of PI4KA depletion by shRNA-mediated knockdown. To this end, Huh7-Lunet/T7-shNT and Huh7-Lunet/T7-shPI4K cells were infected with wt EMCV or each of the two mutants, and at different time points postinfection (p.i.), cells were lysed to determine the total amount of virus by titration. The amount of wt progeny virus was significantly decreased in the PI4KA-knockdown cell line compared to the control cells, particularly at 6 h p.i. and, to a lesser extent, at 8 h p.i., while no such effect was observed for the mutant viruses (Fig. 3B). These results indicated that the 3A mutations completely rescued the delayed replication kinetics observed for wt EMCV.

Next, we asked whether a similar rescue effect could be observed in a single-cycle assay in which PI4KA was inhibited by AL-9 treatment. To this end, HeLa cells were infected with the EMCV wt, EMCV-3A-A32V, or EMCV-3A-A34V strain, treated with dimethyl sulfoxide (DMSO) or AL-9, and lysed at different time points postinfection to determine the total amount of virus by titration. In the presence of AL-9, only the wt virus was significantly inhibited, while the mutants replicated very efficiently throughout infection, to almost the same extent as in the control treatment (Fig. 3C). Dipyridamole, an established inhibitor of EMCV RNA replication (34), completely inhibited the replication of both wt and mutant viruses (Fig. 3C). These results demonstrated that each of the two point mutations in 3A can render EMCV less dependent on PI4KA enzymatic activity.

Previously, we demonstrated that EMCV requires PI4KA activity for the step of RNA genome replication (26). To elucidate if the point mutations in 3A rescue EMCV replication by restoring viral RNA synthesis, we measured the genome replication of the luciferase reporter viruses in the presence of PI4KA inhibitors AL-9 and A1, the latter another PI4KA inhibitor that was recently described (35). Indeed, both substitutions considerably rescued viral RNA replication from the inhibitory activities of both AL-9 and A1 (Fig. 3D).

### PI4KA is recruited to ROs of EMCV mutants.

**FIG 4:**
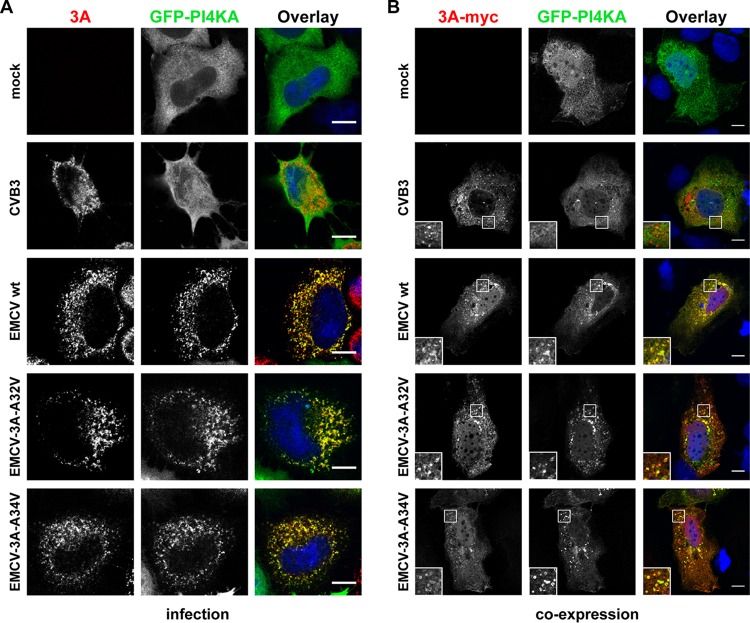
EMCV mutants recruit PI4KA to their ROs. (A) HeLa R19 cells were transfected with a plasmid encoding GFP-PI4KA. The next day, cells were mock infected or infected with CVB3 (included as negative control) or the EMCV wt or mutants at high MOI. At 6 h postinfection (p.i.), cells were fixed and stained with antibodies against EMCV 3AB or CVB3 3A as RO markers. (B) 3A-A32V and 3A-A34V alone can recruit PI4KA to membranes. Huh7-Lunet/T7 cells were cotransfected with the GFP-PI4KA construct and either empty vector or plasmids encoding myc-tagged 3A of CVB3 (included as negative control) or 3A of wt or mutant EMCV. The next day, cells were fixed and stained with antibodies against the myc tag to detect the overexpressed 3A proteins. Insets depict enlargements of boxed areas. (A and B) Nuclei were stained with DAPI (blue). Scale bars represent 10 µm.

Previously, we showed that PI4KA redistributes to EMCV ROs during infection, where it colocalizes with 3A(B) (26). To investigate whether the introduced mutations interfere with PI4KA recruitment by 3A, we examined by immunofluorescence (IF) whether green fluorescent protein-PI4KA (GFP-PI4KA) colocalizes with the mutant 3A proteins, both in the context of virus infection and upon 3A-myc coexpression, using our previously established experimental systems (26). In cells infected with the EMCV wt strain as well as with the EMCV-3A-A32V or EMCV-3A-A34V strain, PI4KA was concentrated at ROs, where it overlapped 3A(B). In contrast, PI4KA did not overlap the 3A signal in CVB3-infected cells, demonstrating the specificity of the recruitment by the EMCV wt and mutants (Fig. 4A). Similarly, upon coexpression, PI4KA was recruited to discrete cytoplasmic 3A-positive puncta by wt or mutant EMCV 3A proteins but not by the 3A of CVB3 (Fig. 4B). Collectively, these data suggested that the mutations introduced in EMCV 3A do not impair PI4KA recruitment to membranes.

### EMCV mutants can replicate in the absence of a PI4P- and cholesterol-enriched environment.

**FIG 5:**
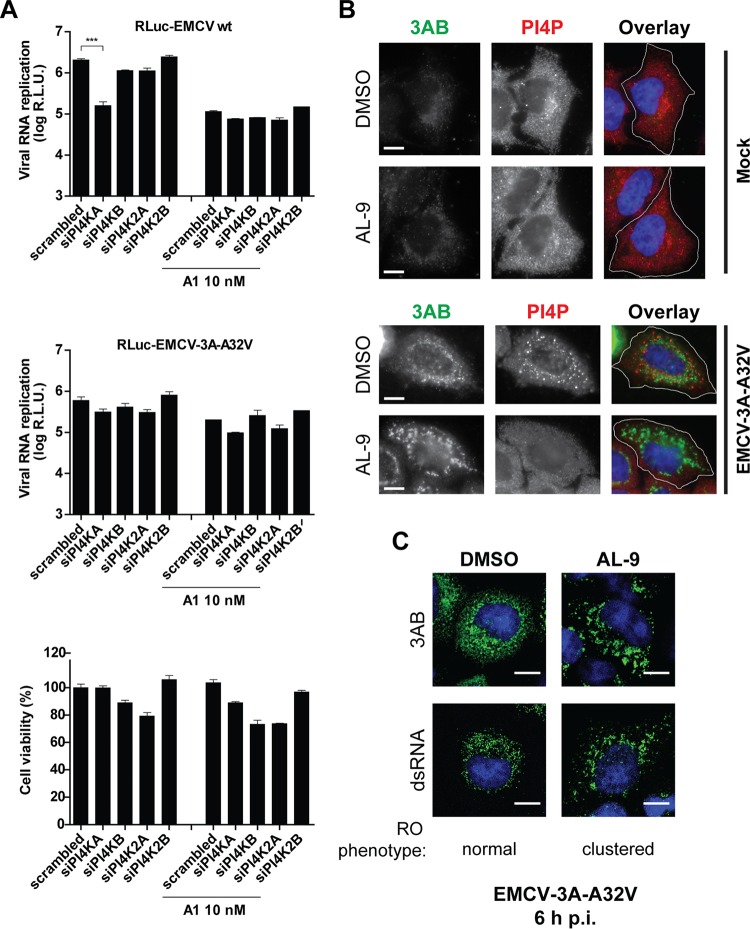
EMCV mutants can replicate in the absence of a PI4P-enriched environment. (A) EMCV-3A-A32V does not rely on other PI4Ks for replication when PI4KA is inhibited. HeLa R19 cells were treated with siRNAs targeting each of the four mammalian PI4Ks. After 48 h, cells were infected with the RLuc-EMCV wt strain or RLuc-EMCV-3A-A32V for 30 min at MOI 1. Medium containing the PI4KA inhibitor A1 (10 nM) was added after removal of the inoculum. Cells were lysed to determine the intracellular luciferase activity after 8 h. In parallel, the cytotoxicity of the siRNA treatment and A1 treatment was determined in a cell viability assay. Mean values ± SEM are shown. Means were statistically compared to the corresponding scrambled siRNA sample using one-way ANOVA. R.L.U., relative light units. (B) HeLa R19 cells were infected with EMCV-3A-A32V for 30 min at MOI 10, followed by treatment with 10 µM AL-9 where indicated. At 6 h p.i., cells were fixed and stained for 3AB and PI4P. For clarity, panels depict the outline of the cells (white line). (C) PI4KA inhibition induces clustering of ROs in mutant EMCV-infected cells. HeLa R19 cells were infected and treated as described for panel B, followed by staining for either 3AB or dsRNA. (A and B) Nuclei were stained with DAPI (blue). Scale bars represent 10 µm.

PI4KA plays an essential role in EMCV replication by inducing a local increase in PI4P, necessary for the downstream accumulation of OSBP and cholesterol at RO membranes (26). We next investigated whether the EMCV mutants might still use a PI4P-dependent pathway in which the PI4P lipids are synthesized by other PI4K isoforms when PI4KA is inhibited. We reasoned that the mutants might use PI4KA in the same manner as the wt virus in the absence of PI4KA inhibitors, while in the presence of PI4KA inhibitors the mutants might switch to using another PI4K isoform. To explore this possibility, we measured the replication of EMCV-3A-A32V in cells depleted of PI4K2A, PI4K2B, or PI4KB by small interfering RNA (siRNA) silencing, using a set of siRNAs previously tested and validated for efficiency in this cell line (9, 21). Two days after the siRNA treatment, cells were infected with either wt or mutant virus in the absence or presence of the PI4KA inhibitor A1. wt EMCV was significantly inhibited only upon PI4KA knockdown, as we showed previously (26), and not upon knockdown of the other PI4K isoforms (Fig. 5A). As expected, EMCV-3A-A32V was resistant to PI4KA knockdown and was not impaired by knockdown of the other isoforms, regardless of the A1 treatment, indicating that the mutants do not rely on PI4P lipids produced by another PI4K when PI4KA activity is impaired.

To verify by a different method that the EMCV 3A mutants do not rely on PI4P lipids, we examined by IF whether these viruses induce PI4P-rich membranes in the presence of a PI4KA inhibitor. Cells were infected for 30 min with virus, after which the virus-containing medium was replaced with fresh medium containing DMSO or AL-9. At 6 h p.i., cells were fixed and analyzed by IF for the presence of viral proteins and PI4P. In mock-infected cells, AL-9 treatment did not affect the intracellular pool of PI4P lipids (Fig. 5B, upper panels), as these are mainly produced by the Golgi complex-localized PI4KB (13–15). In cells infected with the mutants, we detected a strong PI4P signal, distributed throughout the cytoplasm and at 3A-positive structures (Fig. 5B, lower panels). In contrast and despite high levels of viral protein being present, the PI4P signal was hardly detected in cells treated with AL-9 throughout the infection with the mutant, likely due to inhibition of PI4KA activity by AL-9 (Fig. 5B, lower panels). These cells also lacked an apparent Golgi complex PI4P signal, in line with our previous observations that EMCV induces disruption of Golgi complex membranes (26). Under similar conditions, we could not detect cells infected with wt EMCV, since AL-9 blocks its replication (data not shown). Importantly, AL-9 treatment also had a profound impact on the subcellular distribution of the ROs, as revealed by the aggregation of 3A-positive structures into large cytoplasmic clusters that contrast with the small 3A-positive punctate structures typically found in control-treated cells (Fig. 5B; see lower 3AB DMSO and AL-9 panels). The EMCV-3A-A34V mutant displayed a similar phenotype (data not shown). A similar clustering effect induced upon PI4KA inhibition was also observed on the cytoplasmic distribution of viral double-stranded RNA (dsRNA), a marker of viral replication sites (Fig. 5C). Taken together, these findings argued that, in contrast to the wt virus, the mutants no longer necessitated elevated levels of PI4P to establish infection.

**FIG 6:**
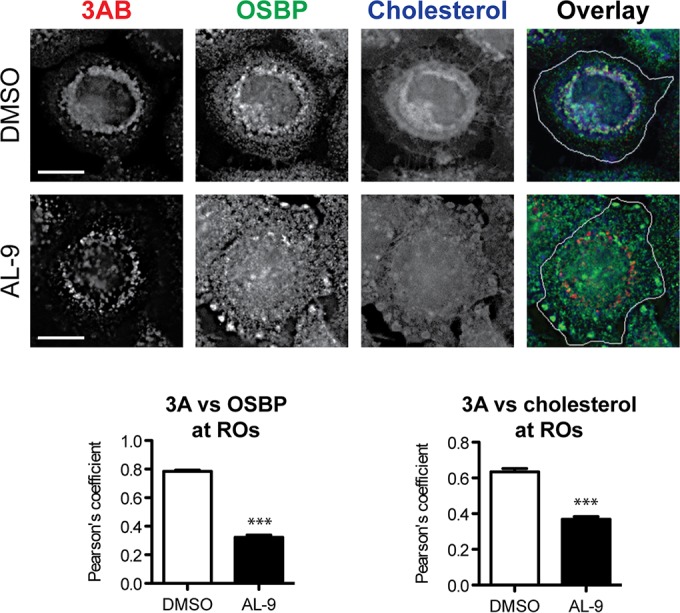
EMCV mutants do not accumulate OSBP and cholesterol at the ROs in the presence of PI4KA inhibitors. HeLa R19 cells were infected with EMCV-3A-A32V for 30 min at MOI 10, followed by treatment with PI4KA inhibitor AL-9 where indicated. At 6 h p.i., cells were fixed and stained for 3AB, OSBP, and cholesterol. Cholesterol was visualized using filipin, a fluorescent antibiotic that specifically binds free cholesterol. For clarity, the overlay panels depict the outline of the cell (white line). Colocalization of OSBP or cholesterol with 3AB was determined by calculating the Pearson’s correlation coefficients for at least 15 cells for each condition. Mean values ± SEM are shown. Means were statistically compared using the Mann-Whitney test. ***, *P* < 0.001. Scale bars represent 10 µm.

Since PI4P lipids serve to accumulate OSBP and cholesterol at EMCV wt ROs (26), we next addressed the issue of whether OSBP and cholesterol were present at the ROs of EMCV 3A mutants when PI4KA was inhibited throughout infection. Cells were infected for 30 min with virus, after which the virus-containing medium was replaced with fresh medium containing DMSO or AL-9. At 6 h p.i., cells were fixed and subjected to IF analysis for the detection of viral 3AB, OSBP, and cholesterol. OSBP and cholesterol colocalized with 3AB at the ROs in infected cells treated with DMSO (Fig. 6, upper panels). The AL-9 treatment significantly reduced the colocalization of 3AB with both OSBP and cholesterol at ROs, which were now also dispersed in patches throughout the cell and close to the plasma membrane (Fig. 6, lower panels). The same phenotypes were observed for the EMCV-3A-A34V mutant (data not shown). These data indicated that the EMCV mutants do not require high levels of OSBP or cholesterol at their ROs to establish infection.

### The 3A mutations provide only minimal resistance to OSBP inhibition.

**FIG 7:**
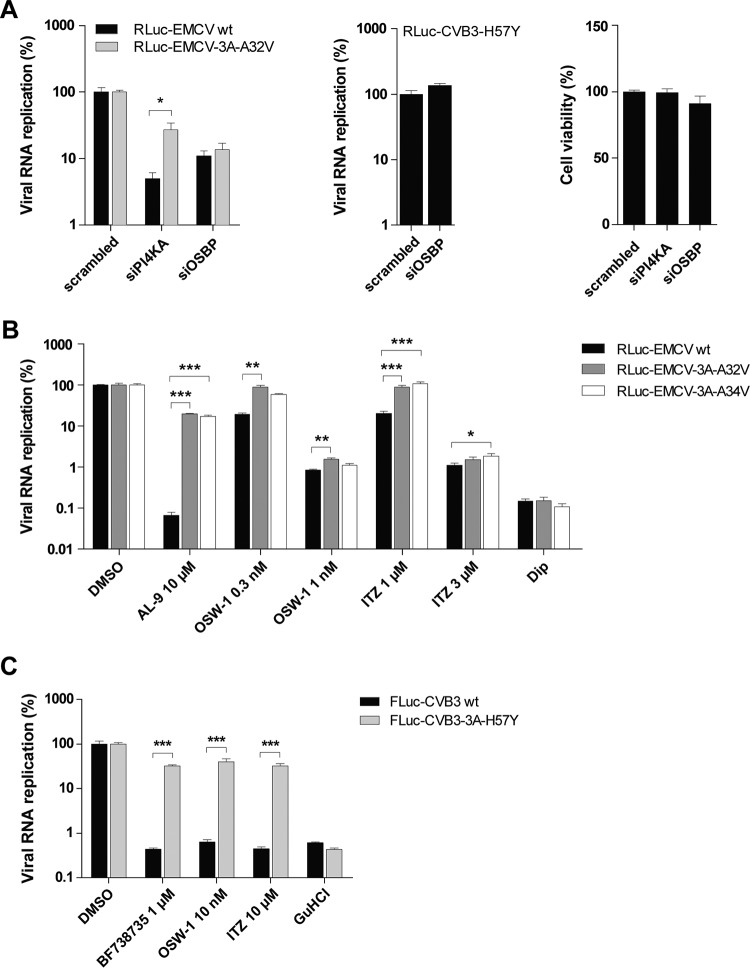
EMCV mutants remain largely sensitive to OSBP depletion or inhibition. (A) Effects of OSBP knockdown on EMCV-3A-A32V. Following treatment with siRNAs for 2 days, HeLa R19 cells were infected with wt EMCV or mutant RLuc-EMCV at MOI 1. After 8 h, cells were lysed to determine the intracellular luciferase activity (left panel). In parallel, off-target effects of the OSBP siRNA treatment were excluded by monitoring replication of the mutant RLuc-CVB3-3A-H57Y (middle panel), previously shown to be resistant to OSBP inhibition (16). Cytotoxicity of the siRNA treatment was determined in a cell viability assay (right panel). (B and C) Sensitivity of wt or mutant EMCV to OSBP inhibitors compared to enteroviruses. HeLa R19 cells were transfected with *in vitro*-transcribed RNA of the EMCV infectious clones encoding *Renilla* luciferase (B) or with *in vitro*-transcribed RNA of the CVB3 subgenomic replicons encoding firefly luciferase in place of the capsid-coding region (C). After RNA transfection, cells were treated with DMSO or with AL-9 or the OSBP inhibitor OSW-1 or itraconazole (ITZ) (B) or with the PI4KB inhibitor BF738735 and OSW-1 or ITZ (C). Guanidine hydrochloride (GuHCl), an established inhibitor of enterovirus replication, was included as a control in the experiments whose results are presented in panel C. Viral RNA replication was determined as described for panel A. The value for the DMSO samples was set at 100%. Mean values ± SEM are shown in all panels. Means were statistically compared using unpaired *t* tests. *, *P* < 0.05; **, *P* < 0.01; ***, *P* < 0.001.

Since the EMCV mutants can replicate without causing apparent enrichment of PI4P, cholesterol, and OSBP at their ROs, we wondered whether they also exhibit a diminished dependence on OSBP. To investigate this, we first compared the sensitivities of EMCV wt and EMCV-3A-A32V to PI4KA versus OSBP knockdown, using a set of siRNAs previously tested and validated for efficiency in this cell line (16, 26). As expected, the mutant replicated considerably more efficiently than the wt in cells depleted of PI4KA. In contrast, OSBP knockdown inhibited the two viruses to similar extents (Fig. 7A, left panel). To exclude potential off-target effects of the OSBP siRNA treatment, we tested in parallel the sensitivity of the mutant enterovirus CVB3-3A-H57Y, previously demonstrated to be resistant to both PI4KB and OSBP inhibition (16, 20, 21, 25). Replication of the CVB3 mutant was not affected by OSBP knockdown (Fig. 7A, middle panel), thus validating the specificity of the observed effects on EMCV replication. No cytotoxicity of the siRNA treatment was observed in the cell viability assay (Fig. 7A, right panel).

Additionally, we measured the effects of different OSBP inhibitors on viral RNA synthesis. To this end, cells were transfected for 1 h with *in vitro*-transcribed genomic viral RNA carried by the RLuc-EMCV wt or each of the mutants, followed by treatment with either AL-9 (as control) or increasing concentrations of the OSBP inhibitor OSW-1 (36) or ITZ (16). The mutants were strongly resistant to AL-9 treatment but remained largely sensitive to OSBP inhibition, with only weak resistance observed when low concentrations of OSW-1 or ITZ were used (Fig. 7B). As a comparison, we tested in parallel the sensitivity of the CVB3-3A-H57Y mutant enterovirus. As expected, replication of CVB3-3A-H57Y was similarly resistant to the PI4KB inhibitor BF738735 and either OSW-1 or ITZ treatment (Fig. 7C). Collectively, these data suggested that, unlike that of the CVB3 3A mutants, the replication of EMCV 3A mutants was still largely dependent on OSBP.

## DISCUSSION

Viruses heavily depend on the cellular machinery and resources to accomplish virtually every step of their replication cycle. In particular, (+)RNA viruses redirect the cellular lipid metabolism toward building specialized membranous replication sites for viral RNA genome synthesis, known as replication organelles (ROs) in the case of picornaviruses (1, 2). Recently, we reported that the picornavirus EMCV relies on the activities of PI4KA and OSBP for RNA genome replication and formation of PI4P- and cholesterol-enriched ROs (26). Here, we describe the selection and characterization of mutant EMCV variants that are less sensitive than the wt to inhibition of PI4KA activity. These EMCV mutants were selected in cells stably depleted of PI4KA by shRNA-mediated knockdown in which replication of wt virus was significantly delayed. Genomic analysis of two different virus pools with accelerated virus growth revealed two independently acquired single-point mutations in the viral 3A protein: A32V and A34V. Introduction of each of these mutations in the wt virus rescued virus replication upon PI4KA knockdown or pharmacological inhibition. In the absence of PI4KA inhibitors, EMCV 3A mutants accumulated PI4P, OSBP, and cholesterol at their ROs, as we have also shown recently for wt EMCV. The mutants did not resort to using another PI4K isoform and did not accumulate PI4P, OSBP, or cholesterol at ROs when PI4KA was inhibited. Furthermore, PI4KA inhibition resulted in aggregation of 3A into large cytoplasmic clusters, indicating that accumulation of PI4P and cholesterol is important for the overall organization of the ROs. Notably, the mutants remained largely sensitive to OSBP inhibition or knockdown, indicative of uncoupled levels of dependency on PI4KA and OSBP.

The PI4KA-OSBP pathway is likewise exploited by HCV (27), which belongs to the distantly related (+)RNA virus family *Flaviviridae*. A previous report by Bianco et al. disclosed unsuccessful attempts to select HCV replicons resistant to the AL-9 PI4KA inhibitor (28). This is in agreement with our repeated failure to select for EMCV mutants by resistance culturing in the presence of AL-9, which might reflect the fact that AL-9 is not a suitable compound for resistance culturing. The isolation of a mutant HCV replicon that was slightly resistant (20-fold compared to wt) to PI4KA inhibition was reported in only one previous study of this kind (37). Genetic analysis of the resistant clone identified several mutations distributed throughout the HCV nonstructural region, but a detailed assessment of these mutations with respect to resistance to PI4KA inhibitors was hampered by impaired replication of the recombinant mutant HCV replicon. Accumulating evidence suggests an additional role of PI4KA in HCV replication through the regulation of NS5A phosphorylation (38–40), a phenomenon that seems to be independent of the need for high levels of PI4P. The complex role of PI4KA in HCV replication likely imposes a high genetic barrier to the development of resistance to PI4KA inhibitors, thereby hampering the generation of viable, PI4KA-independent HCV mutants that could be used to dissect the role of PI4KA in the biogenesis of viral replication membranes. Given the similarities between EMCV and HCV with respect to host requirements for virus genome replication, further characterization of the EMCV mutants described here may be insightful also for understanding the role of PI4P in the context of HCV infection.

Our results indicate that, by acquiring single-point mutations in the 3A protein, EMCV can replicate without an apparent accumulation of PI4P lipids at the ROs. A similar phenotype was previously described for 3A mutant enteroviruses, which also use their 3A protein to hijack the Golgi complex-localized PI4KB (8). Independent single-point mutations in the 3A of CVB3 (e.g., V45A, I54F, and H57Y) and PV (A70T) render these enteroviruses resistant to various PI4KB inhibitors (19–23). While the considerable difference between the amino acid sequences of 3A proteins of cardioviruses and enteroviruses is common knowledge, prior characterization, together with our bioinformatics analysis, suggests that 3A proteins of the genera *Enterovirus*, *Cardiovirus*, and *Senecavirus* may adopt alpha-alpha folds, with the N-terminal and C-terminal domains being soluble and transmembrane domains, respectively. Mutations A32V and A34V in EMCV 3A were predicted to be located at the solvent-exposed surface of the coiled-coil and could therefore act, directly or indirectly, as putative determinants of intermolecular interactions between 3A and other viral and/or host factors, e.g., PI4KA. Likewise, mutations V45A, I54F, and H57Y in CVB3 3A are located in the soluble domain and may also, in theory, modulate intermolecular interactions between 3A and either PI4KB or another factor(s). Another possible mechanism by which these 3A mutations could modulate 3A functionality is by affecting the interaction of 3A with the RO membranes. The latter phenomenon is partially explained by the characteristics of PV 3A mutant A70T, which contains the amino acid change within the 3A hydrophobic membrane anchor (amino acids [aa] 61 to 82). Whether virus replication is rescued by a mechanism that involves a modified interaction of 3A with PI4K or with membranes remains to be determined.

Recently, we demonstrated that acute treatment with PI4KA or OSBP inhibitors impairs the accumulation of PI4P and cholesterol at EMCV ROs and leads to pronounced alterations in the subcellular distribution of the ROs, as revealed by the formation large of 3A(B)-positive clusters (26). This “clustered” phenotype of EMCV ROs highly resembles the NS5A-positive clusters previously observed in HCV replicon cells, also occurring upon PI4KA or OSBP inhibition (and possibly reflecting further similarities shared by EMCV and HCV with respect to the organization of viral replication sites) (27, 28, 38, 41). Here, we observed that prolonged PI4KA inhibition induced a similar RO clustering for the EMCV 3A mutants, as shown by the presence of large, 3A(B)-positive structures in the cytoplasm of infected cells. These 3A(B) clusters also lacked an apparent enrichment in PI4P and cholesterol. Importantly, we observed a similar clustering of the dsRNA signal upon PI4KA inhibition, suggesting that these altered ROs are indeed the sites where viral RNA replication takes place. The clustered structures of wt EMCV and HCV seem to directly correlate with impaired viral RNA replication, but, remarkably, they do not interfere with the replication of EMCV 3A mutants. Two studies demonstrated that the NS5A-positive clusters observed by IF correspond to modifications of the HCV MW at the ultrastructural level (27, 41). Whether these observations also apply to the EMCV remains to be determined.

Apart from contributing to the biogenesis and/or integrity of the ROs, increasing evidence from the enterovirus field suggests an additional role of PI4P and cholesterol in modulating the correct processing of the viral polyprotein. Acute cholesterol depletion was shown to impact the processing of the precursor proteins in CVB3-infected cells (42). Moreover, the A70T mutation in PV 3A was recently suggested to rescue virus replication from PI4KB inhibitors by restoring correct polyprotein processing (43, 44). It remains to be established whether cardioviruses also depend on the lipid microenvironment for efficient polyprotein processing and, if so, whether the A32V or A34V single-point mutation can rescue processing in the presence of PI4KA inhibitors.

Both cardioviruses and enteroviruses induce a high-PI4P microenvironment at the ROs to locally concentrate OSBP, which then shuttles cholesterol to ROs in a PI4P-specific manner (10, 16, 17, 26). The mutations in 3A that render enteroviruses resistant to PI4KB inhibitors also confer strong cross-resistance to OSBP inhibitors (16, 24, 25), providing genetic evidence of the coupled dependency of enteroviruses on PI4KB and OSBP. In contrast to the enterovirus mutants, the EMCV mutants described in this report remained largely sensitive to OSBP inhibition, thus showing apparently uncoupled levels of dependency of virus replication on PI4KA and OSBP. The strong sensitivity of the EMCV mutants to OSBP inhibition compared to PI4KA inhibition points to a noncanonical function of OSBP in virus replication, likely not related to PI4P/cholesterol homeostasis at the RO-MCSs. Thus, the EMCV mutants described here may represent unique tools for investigation of the individual roles of PI4KA and OSBP in (virus-induced) membrane biogenesis.

What could account for the dissimilar sensitivities of the enterovirus versus cardiovirus mutants to OSBP inhibition? The answer to this question awaits further research, but the way mutants were selected could be of relevance. In all studies reported so far, virus was propagated in the cells where PI4K activity was targeted, either by using PI4K(B) inhibitors in the case of enteroviruses (20, 22, 23) or through stable knockdown of PI4K(A) expression for cardioviruses. Thus, upon selection, EMCV needed to adapt not only to shortages of PI4P but also to low PI4KA protein levels, which may have influenced the nature of the escape mutations. Another explanation for the distinct resistance profiles of the mutants could reside in their differential host cell requirements. As shown here and in previous studies (16, 26), wt EMCV seems to be more sensitive than wt CVB3 to OSBP inhibition, corroborating the distinct resistance phenotypes of the mutant viruses. Further divergence in host factor usage is also reflected in the fact that enteroviruses mainly target Golgi complex membranes and factors (45), whereas cardioviruses rely instead on ER-derived membranes (26, 46). Finally, our study results prove that genetically and phenotypically divergent picornaviruses can acquire mutations in sequence-dissimilar 3A proteins to replicate efficiently without inducing enrichment of PI4P at ROs, highlighting the outstanding adaptability of picornaviruses to changing environmental conditions.

## MATERIALS AND METHODS

### Cells and reagents.

Buffalo green monkey (BGM), baby hamster kidney 21 (BHK-21), and HeLa R19 cells were maintained at 37°C under 5% CO_2_ in Dulbecco’s modified Eagle’s medium (DMEM; Lonza) supplemented with 10% fetal bovine serum (FBS). Huh7-Lunet/T7 cells (provided by R. Bartenschlager, Department of Molecular Virology, University of Heidelberg, Heidelberg, Germany) (47) were grown in DMEM (Lonza) supplemented with 10% FBS and 10 µg/ml blasticidin (phosphonoacetic acid [PAA]). For the stable cell lines Huh7-Lunet/T7-shNT and Huh7-Lunet/T7-shPI4KA (29), 2 µg/ml puromycin and 5 µg/ml zeocin (Invitrogen) were added to the culture medium. Huh7-Lunet/T7-shPI4KA cells stably express short hairpin RNA (shRNA) for knockdown of PI4KA, while Huh7-Lunet/T7-shNT is the corresponding control cell line expressing nontargeting short hairpin RNA. OSW-1 was generously provided by M. D. Shair (Department of Chemistry and Chemical Biology, Harvard University, Cambridge, MA, USA). A1 (35) was a kind gift from T. Balla (National Institutes of Child Health and Human Development, National Institutes of Health, Bethesda, MD, USA). BF738735 (20, 48) was provided by Galapagos NV. Itraconazole was purchased from Santa Cruz Biotechnology. Filipin III, dipyridamole (Dip), and guanidine hydrochloride (GuHCl) were from Sigma. All compounds were dissolved in DMSO, except for dipyridamole, which was dissolved in ethanol.

### Plasmids.

Plasmids pEGFP-PI4KA (kindly provided by G. Hammond, NICHD, National Institutes of Health, Bethesda, MD, USA) (49, 50), p3A-myc (CVB3) (51), and p3A-myc (EMCV) (26) were described previously. Plasmid pM16.1 contains the full-length infectious cDNA sequence of EMCV, strain mengovirus. Plasmid pRLuc-QG-M16.1 was obtained by introducing the *Renilla* luciferase gene upstream of the capsid coding region (52) in pM16.1. Infectious clones EMCV-3A-A32V and RLuc-EMCV-3A-A32V were generated by introducing the A32V mutation into the 3A sequence of pM16.1 and pRLuc-QG-M16.1, respectively, using mutagenesis primers designed with the NEBaseChanger tool and a Q5 site-directed mutagenesis kit (New England Biolabs) according to the manufacturer’s instructions. EMCV-3A-A34V and RLuc-EMCV-3A-A34V were generated as described for the 3A-A32V mutants. Constructs p3A-A32V-myc and p3A-A34V-myc were generated from p3A-myc (EMCV) using the same strategy. Subgenomic replicons pRib-LUC-CB3/T7 and pRib-LUC-CB3/T7-3A-H57Y, encoding Firefly luciferase in place of the capsid-coding region, were described previously (19, 53).

### Virus infection and replicon assays.

All EMCV and RLuc-EMCVs were obtained by transfecting BHK-21 cells with RNA transcripts derived from their respective full-length infectious clones linearized with BamHI. CVB3 (strain Nancy) and RLuc-CVB3-3A-H57Y were obtained by transfecting BGM cells with RNA transcripts of the full-length infectious clones p53CB3 (51) and pRLuc-53CB3/T7-3A-H57Y (21), respectively, linearized with SalI. Virus infections were performed by incubating subconfluent cell monolayers for 30 min at 37°C with virus, after which the inoculum was removed and fresh (compound-containing) medium was added to the cells (*t* = 0). Alternatively, cells were transfected with *in vitro*-transcribed RNA of linearized subgenomic replicons or infectious clone RLuc-EMCV wt or mutant, using Lipofectamine 2000 (Invitrogen). One hour later, transfection medium was replaced with fresh (compound-containing) medium. At the indicated time points postinfection or posttransfection (pt), cells were fixed for immunolabeling or freeze-thawed to determine virus titers or lysed to determine intracellular *Renilla* or Firefly luciferase activity using a *Renilla* or Firefly luciferase assay system (Promega), when using the luciferase reporter viruses and replicons. Virus titers were determined by endpoint titration according to the method of Reed and Muench and expressed as 50% tissue culture infective doses (TCID_50_).

### Selection of PI4KA-independent EMCV variants.

PI4KA-independent EMCV mutants were selected by serial passaging of wild-type (wt) EMCV in Huh7-Lunet/T7-shPI4KA cells. This procedure was started by preparing serial dilutions of a wt EMCV stock in 96-well plates containing Huh7-Lunet/T7-shPI4KA cells. The highest virus dilution that induced CPE (10^8^) was subsequently passaged in Huh7-Lunet/T7-shPI4KA cells in 96-well plates, and virus cultures from individual wells were harvested based on early CPE development. Next, two virus cultures (from wells 3E1 and 4E3) were selected and further passaged four times in Huh7-Lunet/T7-shPI4KA cells at a multiplicity of infection of 1 (MOI 1), with supernatants containing mutant viruses harvested each time at 16 h p.i. (prior to full CPE). The obtained virus cultures were then tested for resistance to AL-9. Finally, the viral genomic RNA was isolated, used for cDNA synthesis by reverse transcription-PCR (RT-PCR), and subjected to Sanger sequencing analysis.

### Immunofluorescence microscopy.

HeLa R19 or Huh7-Lunet/T7 cells were grown to subconfluency on coverslips in 24-well plates. Where indicated, cells were transfected with 400 ng of plasmids using Lipofectamine 2000 according to the manufacturer’s protocol and/or mock infected or infected for 30 min with EMCV or CVB3 at the specified multiplicity of infection (MOI), followed by compound treatment where specified. At the indicated time points, cells were fixed with 4% paraformaldehyde for 20 min at room temperature. For filipin staining, permeabilization was done with phosphate-buffered saline (PBS)–0.2% saponin–5% bovine serum albumin (BSA) for 5 min. Staining of intracellular PI4P was performed as described elsewhere (26, 54). Briefly, cells were fixed with 2% paraformaldehyde (PFA), permeabilized for 5 min in 20 µM digitonin–buffer A {20mM PIPES [piperazine-*N*,*N*′-bis(2-ethanesulfonic acid)] [pH 6.8], 137 mM NaCl, 2.7 mM KCl}, blocked for 45 min in buffer A with 5% normal goat serum (NGS) and 50 mM NH_4_Cl, and then incubated sequentially with primary and secondary antibodies in buffer A with 5% NGS, before postfixation in 2% PFA for 10 min. The following primary antibodies were used for detection: mouse monoclonal anti-PI4P IgM (Echelon Biosciences), mouse monoclonal anti-EMCV 3AB (kind gift from A. G. Aminev) (55), mouse monoclonal anti-dsRNA (J2; English and Scientific Consulting), rabbit polyclonal anti-myc (Thermo Scientific), and rabbit polyclonal anti-OSBP (kindly provided by M. A. De Matteis, Telethon Institute of Genetics and Medicine, Naples, Italy) (16). Alexa Fluor 488-and-594-conjugated IgG and Alexa Fluor 594-conjugated IgM (Invitrogen, Molecular Probes) were used as secondary antibodies. Cholesterol was stained during the incubation with the secondary antibody with 25 µg/ml filipin III for 1 h at room temperature. Nuclei were counterstained with DAPI (4′,6-diamidino-2-phenylindole). Coverslips were mounted with FluorSave (Calbiochem). Images were acquired with a Nikon Ti Eclipse microscope equipped with an Andor DU-897 electron-multiplying charge-coupled-device (EMCCD) camera.

### Image analysis.

To quantify colocalization of OSBP and filipin with 3AB, images containing Z-stacks taken at ~100-nm intervals throughout the depth of the cells were first deconvoluted using NIS software (20 iterations) and further processed using ImageJ as follows. Individual infected cells were outlined and a mask was created, and the signal outside the mask was cropped to exclude it from the calculations. The Pearson’s coefficient of colocalization was determined for at least 15 cells per condition using the Coloc 2 plugin with default settings.

### siRNA treatment.

HeLa R19 cells were reverse transfected with 2 pmol of siRNA per well of a 96-well plate (2,000 cells/well) using Lipofectamine 2000 (Invitrogen) according to the manufacturer’s indications. Scrambled siRNA (AllStars Negative Control; Qiagen) was used as a control. siRNA against hPI4KA (catalog no. S102777390) and hPI4KB (target sequence, 5′-UGUUGGGGCUUCCCUGCCCTT-3′) were from Qiagen. siRNA against human OSBQ (hOSBP) (two siRNAs mixed at a 1:1 ratio; target sequences, 5′-CGCUAAUGGAAGAAGUUUA[dT][dT]-3′ and 5′-CCUUUGAGCUGGACCGAUU[dT][dT]-3′) was from Sigma. siRNA against PI4K2A and siRNA against PI4K2B were from Ambion (21). At 48 h pt, cells were either infected with virus or analyzed in a cell viability assay.

### Cell viability assay.

Cell viability was determined in parallel with virus infection. The medium was replaced with CellTiter 96 AQueous One solution reagent (Promega), and optical densities were measured at 490 nm following a 2-h incubation at 37°C under 5% CO_2_. The obtained raw values were converted to percentages of samples transfected with scrambled siRNAs, after correction for background absorbance.

### Statistical analyses.

Where indicated, an unpaired one-tailed Student’s *t* test, one-way analysis of variance (ANOVA), or a two-tailed Mann-Whitney test was applied for statistical analysis using GraphPad Prism software.

### Bioinformatics analyses.

Multiple-sequence alignments (MSA) of 3A proteins of cardio- and senecaviruses were generated using the Viralis platform (56) assisted by the use of HMMER 3.1 (57), muscle 3.8.31 (58), and ClustalW 2.012 (59) programs in default modes. A subset of this MSA, including one virus per species (in total, four sequences), was then prepared using GeneDoc 2.7 (60) to highlight conservation. This MSA and/or its separate sequences were used as the input to predict the secondary protein structure by the use of PsiPred (30), transmembrane domains by the use of TMHMM2 (32), and coiled-coils by the use of Paircoil2 (31).
